# Tectorigenin, a Flavonoid-Based Compound of Leopard Lily Rhizome, Attenuates UV-B-Induced Apoptosis and Collagen Degradation by Inhibiting Oxidative Stress in Human Keratinocytes

**DOI:** 10.3390/nu10121998

**Published:** 2018-12-17

**Authors:** Dongjin Noh, Jin Gyu Choi, Eugene Huh, Myung Sook Oh

**Affiliations:** 1 Department of Life and Nanopharmaceutical Sciences, Graduates School, Kyung Hee University, 26 Kyungheedae-ro, Dongdaemun-gu, Seoul 02447, Korea; djnoh92@khu.ac.kr (D.N.); eugenehuh@khu.ac.kr (E.H.); 2 Department of Pharmacy, College of Pharmacy, Kyung Hee University, 26 Kyungheedae-ro, Dongdaemun-gu, Seoul 02447, Korea; choijg2002@khu.ac.kr; 3 Department of Medical Science of Meridian, Graduates School, Kyung Hee University, 26 Kyungheedae-ro, Dongdaemun-gu, Seoul 02447, Korea; 4 Department of Oriental Pharmaceutical Science, College of Pharmacy and Kyung Hee East-West Pharmaceutical Research Institute, Kyung Hee University, 26 Kyungheedae-ro, Dongdaemun-gu, Seoul 02447, Korea

**Keywords:** tectorigenin, skin damage, ultraviolet light, keratinocytes, antioxidants

## Abstract

Ultraviolet (UV) light, a major risk factor for external skin photoaging, induces oxidative stress in skin. UV causes a breakdown of skin homeostasis by impairing the extracellular matrix and inducing cell death. Tectorigenin, a constituent of leopard lily (*Belamcanda chinensis* L.) rhizome, has been reported to possess antioxidant, hair-darkening, and anti-inflammatory activities; however, the effect of tectorigenin on UV-B-induced skin damage is unknown. Here, we investigated the anti-skin-damage effects of tectorigenin against UV-B-stimulated oxidative stress in human keratinocytes. We irradiated HaCaT cells with UV-B (25 mJ/cm^2^), followed by treatment with tectorigenin for 24 h. We found that tectorigenin decreased the levels of intracellular reactive oxygen species by increasing the expression of anti-oxidative enzymes, such as glutathione and catalase. Furthermore, tectorigenin inhibited apoptosis by reducing caspase-3- and Bcl-2-associated protein-X levels, and increasing Bcl-2 protein levels. Tectorigenin also decreased matrix metalloproteinase-1 levels and increased type 1 collagen levels, thus preventing collagen degradation. These data demonstrate that tectorigenin exerts anti-skin-damage effects in human keratinocytes by attenuating UV-B-induced hyper-oxidation, apoptosis, and collagen degradation.

## 1. Introduction

Physiological aging of the skin includes the formation of wrinkles, pigmentation, and drying. Two types of skin aging are recognized: intrinsic aging, a natural phenomenon that occurs as humans age, and external aging, which is caused by exposure to the external environment [1]. External aging is mediated by external factors, such as solar ultraviolet-A (UV-A), UV-B, and air pollution. They generate reactive oxygen species (ROS) in the skin [2]. UV-A, a long wavelength of ultraviolet (315–400 nm), is known to penetrate into skin more deeply than UV-B and damages the dermis. UV-B, a medium wavelength of ultraviolet (280–315 nm), damages epidermis by causing direct DNA damage [3]. Air pollutants, such as tobacco smoke and ozone, also damage skin by generating ROS in skin cells; however, they are considered to be less important than UV irradiation [4]. Among them, UV-B is considered to be the most crucial factor because it has higher energy than UV-A and causes a breakdown of skin homeostasis. One of the consequences of ROS generation is that Bcl-2-associated protein-X (Bax) levels increase, which leads to a lower mitochondrial membrane potential via the activation of mitochondrial-voltage-dependent anion channels. Bax also induces the release of cytochrome c in mitochondria and activates caspase-3, which results in cell death [5]. In addition, UV-B irradiation promotes the expression of matrix metalloproteinase-1 (MMP-1), a major collagenase that breaks down type 1 collagen, resulting in a loss of skin tension and, therefore, wrinkle formation [6,7].

The skin has several defense mechanisms to protect it from oxidative damage and maintain its normal physiological condition. The former include anti-oxidative enzymes, such as superoxide dismutase (SOD), catalase, and glutathione (GSH), which neutralize intracellular ROS [8]. On the other hand, there is considerable evidence that natural compounds from botanical products have an anti-oxidative effect.

Phytochemicals are derived from natural products, and have long been used to treat a variety of diseases, such as psoriasis and atopic dermatitis [9,10]. These plant-derived compounds are easy to harvest and, based on their antioxidant properties, their applications include the attenuation of skin photoaging. For example, (−)-epigallocatechin-3-gallate, a flavanol found in green tea, protects hairless mouse skin and human dermal fibroblasts against UV-induced skin damage [6]. Quercetin, a flavonol present in high concentrations in red onions, protects human keratinocytes against UV-induced inflammatory cytokine production by inhibiting the induction of nuclear factor κ-B [11]. These reports suggest that other, as yet undescribed, flavonoids will have protective effects on UV-B-induced skin damage.

**Figure 1:**
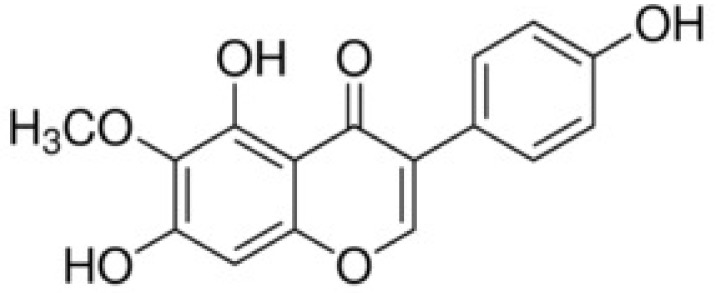
The chemical structure of tectorigenin.

Tectorigenin, an O-methylated isoflavone, is a constituent of leopard lily (*Belamcanda chinensis* L.) rhizome (Figure 1). Its antioxidant effects have been attributed to the induction of anti-oxidative enzyme expression. For example, tectorigenin was shown to attenuate 1-methyl-4-phenylpyridinium-induced oxidative stress in SH-SY5Y cells by upregulating the SOD, catalase, and GSH peroxidase level [12]. In the rat liver, tectorigenin ameliorated fibrosis by increasing SOD and GSH peroxidase [13]. Therefore, in this study, we investigated the ability of tectorigenin to prevent UV-B-induced damage to keratinocytes: (1) by upregulating the expression of the antioxidant enzymes GSH and catalase; (2) by inhibiting keratinocyte apoptosis, as determined by reductions in the levels of the apoptotic proteins Bcl-2, Bax, and caspase-3; and (3) by preventing MMP-1-mediated collagen degradation.

## 2. Materials and Methods

### 2.1. Materials

Tectorigenin was obtained from Chemfaces (Hubei, China). Dulbecco’s modified Eagle’s medium (DMEM), fetal bovine serum (FBS), and penicillin-streptomycin were purchased from Hyclone Laboratories, Inc. (Auckland, New Zealnad). 3-(4,5-Dimethylthiazol-2-yl)-2,5-diphenyltetrazolium bromide (MTT), hydrogen peroxide, and 2′,7′-dichlorodihydrofluorescein diacetate (DCFH-DA) were purchased from Sigma-Aldrich (St. Louis, MO, USA). Protein standards dual color, a western view marker, a protein assay, tween-20, acrylamide, ammonium persulfate, skim milk, and enhanced chemiluminescence (ECL) reagent were purchased from BioRad Laboratories (Hercules, CA, USA). Dimethyl sulfoxide (DMSO) was obtained from Junsei (Tokyo, Japan). A total glutathione quantification kit was purchased from Dojindo (Kumamoto, Japan). An MMP-1 ELISA kit was purchased from R&D systems (Minneapolis, MN, USA). Rabbit anti-Bax was obtained from Abcam (Cambridge, UK). Horseradish peroxidase (HRP) anti-β-actin Antibody, rabbit anti-catalase, and rabbit anti-Bcl-2 were obtained from Santa Cruz Biotechnology (Dallas, TX, USA). Rabbit anti-caspase-3 was purchased from Cell Signaling Technology (Danvers, MA, USA). Rabbit anti-Col1a1 was obtained from Thermo Fisher Scientific (San Jose, CA, USA). HRP secondary antibody was purchased from Enzo Life Sciences Inc. (Farmingdale, NY, USA). Protein extraction buffer was obtained from Intron biotechnology (Seongnam, Korea). Phosphate-buffered saline (PBS) was obtained from Abcam (Cambridge, MA, USA). The other reagents used were of guaranteed or analytical grade.

### 2.2. Cell Culture

Cells from the HaCaT human epidermal keratinocytes cell line were kindly donated by Prof. Sun Yeou Kim at Gachon University, Korea. Cells were maintained in DMEM supplemented with 10% heat-inactivated FBS and 1% penicillin/streptomycin under conditions of 95% air and 5% CO_2_ at 37 °C.

### 2.3. Measurement of Cell Viability

An MTT assay was performed to measure the toxicity and protective effects of tectorigenin in HaCaT cells. MTT is commonly used to measure cell viability because it produces purple formazan in response to mitochondrial enzymes in live cells [14]. Cells were seeded in 96-well plates at 1.0 × 10^4^ cells/well and incubated for 24 h. Next, cells were washed 3 times with PBS and UV-B was irradiated at 25 mJ/cm^2^. UV-B irradiation was supplied by a closely spaced array of 5 Sankyo Denki sunlamps, which delivered uniform radiation at a distance of 7.5 cm. After cells were washed 3 times and treated with tectorigenin or vitamin C, cells were incubated for 24 h. After removing the supernatant, 1 μg/mL MTT was added and incubated for 4 h. After removing the supernatant, 100 μL of DMSO was added to formazans and the absorbance was measured at 570 nm.

### 2.4. Measurement of Intracellular ROS

DCFH-DA fluorescence was used to measure intracellular ROS in HaCaT cells. Cells were seeded in 96-well black plates at 1.0 × 10^4^ cells/well or on coverslips in 24-well plates at 2.0 × 10^4^ cells/well and incubated for 24 h. Then, the medium was replaced with serum-free DMEM and cultured for 24 h. Next, cells were washed 3 times with PBS, and UV-B was irradiated at 25 mJ/cm^2^. After cells were washed 3 times and treated with tectorigenin or vitamin C, cells were incubated for 30 min. After removing the supernatant, 20 μM of DCF-DA was added. After incubation for an additional 30 min, cells were washed 3 times with PBS and the fluorescence was measured at 485 nm excitation and 535 nm emission using a fluorescence microplate reader (SpectraMax Gemini EM, Molecular Device; Sunnyvale, CA, USA). Representative images were taken by a fluorescence microscope (Olympus Microscope System BX51; Olympus, Tokyo, Japan).

### 2.5. Western Blot Analysis

Cells were seeded in a 60-mm dish at 1.2 × 10^6^ cells/well and incubated for 24 h. After cells were washed with PBS 3 times, UV-B was irradiated with 5 mJ/cm^2^ for measuring type I collagen levels or 25 mJ/cm^2^ for measuring other protein levels. After cells were washed 3 times and treated with tectorigenin or vitamin C, cells were incubated for 2 h for measuring other protein levels or 24 h for measuring type I collagen levels. The supernatant was removed and the cells were collected and centrifuged to obtain pellets. After the lysis in buffer, the supernatant was obtained by centrifugation and used for the experiment. Protein concentrations were quantified by Bradford’s assay by using the kit according to the manufacturer’s instruction (BioRad, Hercules, CA, USA). Thirty micrograms (30 μg) of protein in each sample were loaded in 12% SDS-acrylamide gel, and then loaded proteins were electrophoretically transferred to a membrane. β-actin (1:3000), Bcl-2 (1:500), Bax (1:500), caspase-3 (1:500), catalase (1:500), and Col1a1 (1:1000) were incubated in tris buffered saline with Tween 20 with 5% skim milk at 4 °C overnight. Anti-rabbit HRP-conjugated (1:2000) and anti-mouse HRP-conjugated (1:2000) antibodies were used as the secondary antibody for 1 h. Membranes were reacted using an ECL detection kit and band images were obtained by using an LAS-4000 mini system (Fujifilm, Tokyo, Japan). The band intensities were also quantified by using Image J (Bethesda, MD, USA) and compared to the band intensities of β-actin.

### 2.6. Measurement of MMP-1 Levels

Cells were seeded in 24-well plates at 2.0 × 10^4^ cells/well and incubated for 24 h. Next, cells were washed with PBS 3 times and UV-B was irradiated at 5 mJ/cm^2^. After cells were washed 3 times and treated with tectorigenin or vitamin C, cells were incubated for 24 h. The supernatant was collected and the levels of MMP-1 were measured by using an MMP-1 ELISA kit (R&D Systems, Minneapolis, MN, USA).

### 2.7. Measurement of GSH Levels

Cells were seeded in 60-mm dishes at 1.2 × 10^6^ cells/well and incubated for 24 h. Next, cells were washed with PBS 3 times, and UV-B was irradiated at 25 mJ/cm^2^. HaCaT cells were washed 3 times, treated with tectorigenin or vitamin C, and incubated for 2 h. After the cells were harvested, GSH contents were measured by a Total glutathione quantification kit (Dojindo, Kumamoto, Japan).

### 2.8. Statistical Analysis

All statistical parameters were calculated using GraphPad Prism 4.0 software (GraphPad Software Inc., San Diego, CA, USA). Values were expressed as the mean ± standard error of the mean (SEM). The results were analyzed by one-way analysis of variance (ANOVA) and post hoc multiple mean comparisons (Tukey’s honestly significant difference test).

## 3. Results

### 3.1. Tectorigenin Protects Keratinocytes against UV-B-Induced Damage

**Figure 2:**
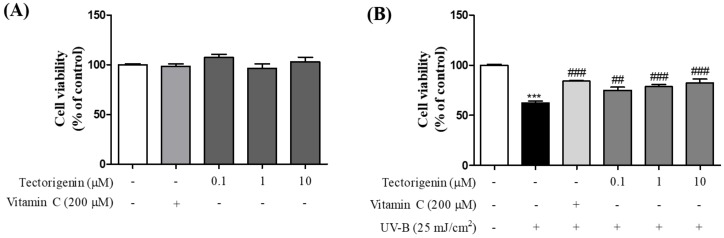
Inhibitory effects of tectorigenin against UV-B-induced cell death in HaCaT cells. The cells were treated with tectorigenin at 0.1, 1, and 10 μM or vitamin C at 200 μM for 24 h (**A**). The cells were irradiated with 25 mJ/cm^2^ UV-B and treated with tectorigenin at 0.1, 1, and 10 μM or vitamin C at 200 μM for 24 h (**B**). Cell viability was measured by an MTT assay. Values are given as the mean ± the standard error of the mean (SEM). *** *p* < 0.001 versus control group, ## *p* < 0.01, ### *p* < 0.001 versus UV-B-only treated group.

MTT assays were performed to determine the cytotoxicity of tectorigenin and the effects of the isoflavonoid against UV-B-induced damage. Tectorigenin at concentrations between 0.1 and 10 μM was not toxic for HaCaT cells (Figure 2A). While UV-B irradiation caused cell damage (62.33 ± 1.81%) compared to the control group, treatment of the cells with tectorigenin significantly inhibited cell death (0.1, 1, and 10 μM; 74.94 ± 3.09%, 78.58 ± 2.28%, and 82.36 ± 3.81%, respectively) compared to the UV-B-only group (Figure 2B).

### 3.2. Tectorigenin Inhibits UV-B-Induced ROS Generation in Keratinocytes

**Figure 3:**
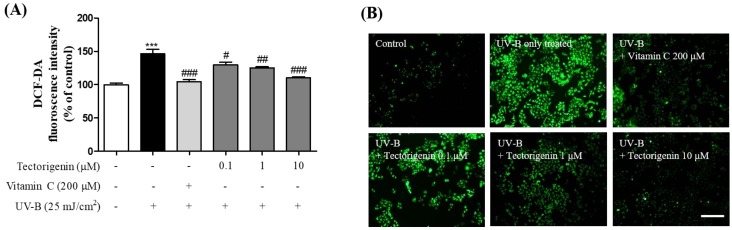
Inhibitory effects of tectorigenin on UV-B-induced generation of intracellular reactive oxygen species (ROS) in HaCaT cells. The cells were irradiated with 25 mJ/cm^2^ UV-B and treated with tectorigenin at 0.1, 1, and 10 μM or vitamin C at 200 μM for 24 h (**A**,**B**). Intracellular ROS were measured by a DCFH-DA assay. Values are given as the mean ± SEM. *** *p* < 0.001 versus control group, # *p* < 0.05, ## *p* < 0.01, ### *p* < 0.001 versus UV-B-only treated group (**A**). Representative photomicrographs are shown. Scale bar = 50 μm (**B**).

The effects of tectorigenin on intracellular ROS generation induced by UV-B irradiation were evaluated in a DCFH-DA fluorescence assay. Intracellular ROS levels were increased by UV-B irradiation compared to the control group (146.44 ± 6.62%), but were significantly reduced by tectorigenin treatment (0.1, 1, and 10 μM; 119.18 ± 2.11%, 113.63 ± 2.46%, and 103.38 ± 2.08%, respectively) compared to the UV-B-only group (Figure 3).

### 3.3. Tectorigenin Stimulates the Release of Anti-Oxidative Enzymes Effective against UV-B-Induced Damage

**Figure 4:**
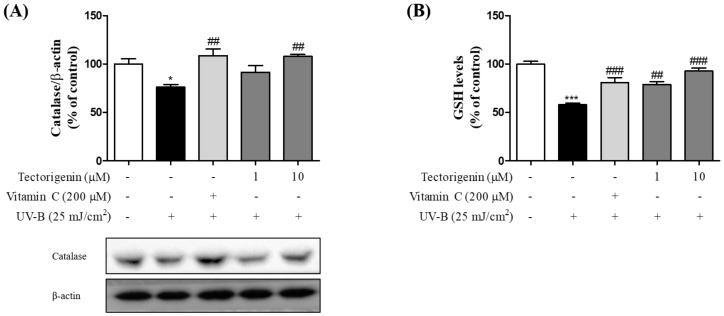
Inhibitory effects of tectorigenin on UV-B-induced downregulation of anti-oxidative enzymes in HaCaT cells. The cells were irradiated with 25 mJ/cm^2^ UV-B and treated with tectorigenin at 1 and 10 μM or vitamin C at 200 μM for 24 h (**A**,**B**). Levels anti-oxidative enzymes were measured by a Western blot analysis. Values are given as the mean ± SEM. * *p* < 0.05, *** *p* < 0.001 versus control group, ## *p* < 0.01, ### *p* < 0.001 versus UV-B-only treated group.

In the UV-B-treated group, catalase levels were markedly lower than in the control group (75.99 ± 2.77%), while in the group treated with tectorigenin (1 and 10 μM), catalase levels were significantly higher (1 and 10 μM; 91.58 ± 6.94% and 107.77 ± 2.05%, respectively) than in the UV-B-only group (Figure 4A). Moreover, UV-B irradiation resulted in a decrease in GSH levels compared to the control group (57.76 ± 1.71%). This loss was significantly inhibited by tectorigenin (1 and 10 µM; 78.81 ± 5.05% and 92.72 ± 3.01%, respectively) (Figure 4B).

### 3.4. Tectorigenin Protects Keratinocytes against the UV-B-Induced Expression of Apoptosis-Related Proteins

**Figure 5:**
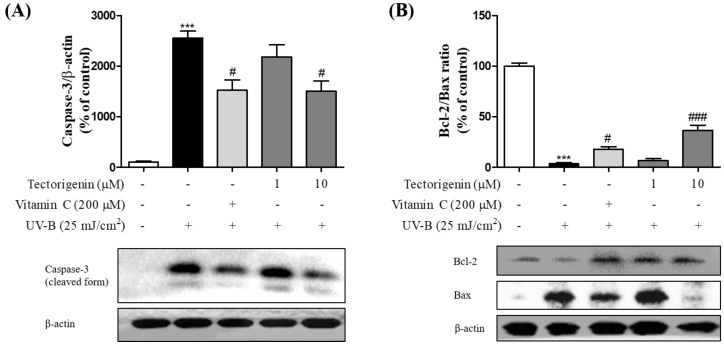
Inhibitory effects of tectorigenin on the UV-B-induced expression of cleaved caspase-3 and downregulation of the Bcl-2/Bax ratio in HaCaT cells. The cells were irradiated with 25 mJ/cm^2^ UV-B and treated with tectorigenin at 1 and 10 μM or vitamin C at 200 μM for 24 h (**A**,**B**). Caspase-3 levels and the Bcl-2/Bax ratio were measured by Western blotting. Values are given as the mean ± SEM. *** *p* < 0.001 versus control group, # *p* < 0.05, ### *p* < 0.001 versus UV-B-only treated group.

UV-B-induced apoptosis-related protein expression was significantly diminished by tectorigenin (1 and 10 μM), as measured by reductions in the levels of caspase-3 (1 and 10 μM; 2182.47 ± 236.94% and 1504.54 ± 199.46%, respectively) compared with the UV-B-only treated group (Figure 5A). In the UV-B-only treated group, the Bcl-2/Bax ratio was significantly lower than in the control group (3.48 ± 0.98%); however, the effect was significantly reversed by tectorigenin (1 and 10 μM; 6.31 ± 1.99% and 36.42 ± 5.10%, respectively) (Figure 5B).

### 3.5. Tectorigenin Attenuates UV-B-Induced Collagen Degradation in Keratinocytes

**Figure 6:**
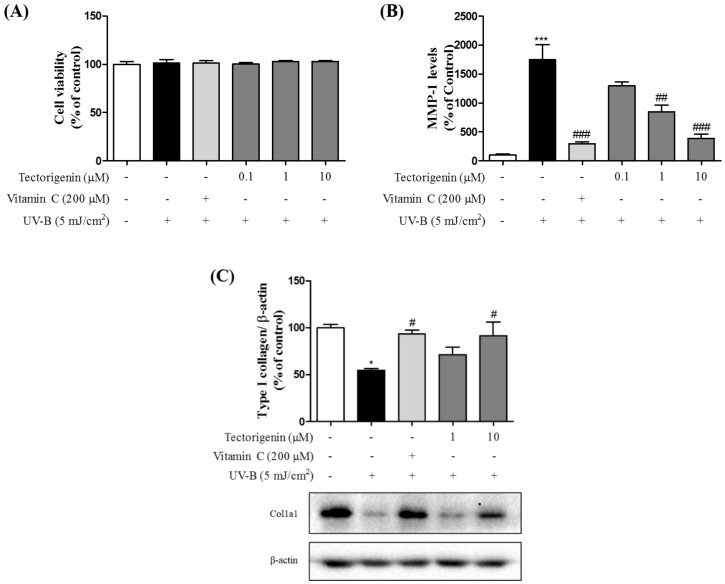
Inhibitory effects and the cytotoxicity of tectorigenin on UV-B-induced expression of MMP-1 and collagen degradation in HaCaT cells. The cells were irradiated with 5 mJ/cm^2^ UV-B and treated with tectorigenin at 0.1, 1, and 10 μM or vitamin C at 200 μM for 24 h (**A**,**B**). The cells were irradiated with 5 mJ/cm^2^ UV-B and treated with tectorigenin at 1 and 10 μM or vitamin C at 200 μM for 24 h (**C**). Cell viability was measured by an MTT assay. MMP-1 levels were measured by a human MMP-1 ELISA kit. Type 1 collagen levels were measured by a Western blot analysis. Values are given as the mean ± SEM. * *p* < 0.05, *** *p* < 0.001 versus control group, # *p* < 0.05, ## *p* < 0.01, ### *p* < 0.001 versus UV-B-only treated group.

Cytotoxicity was not shown in any groups (Figure 6A). Tectorigenin treatment significantly reduced the levels of MMP-1 (0.1, 1, and 10 μM; 1297.55 ± 62.82%, 844.775 ± 115.55%, and 387.97 ± 74.13%, respectively) compared with the UV-B-only group (1747.26 ± 259.15%) (Figure 6B). Additionally, UV-B irradiation resulted in markedly lower levels of type 1 collagen compared to the control group, whereas the effect was ameliorated by tectorigenin (1 and 10 μM; 71.19 ± 7.92% and 91.56 ± 14.57%, respectively) (Figure 6C).

## 4. Discussion

This study investigated the effects of tectorigenin on UV-B-induced keratinocyte apoptosis and collagen degradation. The results demonstrated the potent protective effects of tectorigenin against UV-B-induced skin damage.

UV-B is a major cause of skin photoaging, an effect mediated by the generation of ROS, such as superoxides, hydrogen peroxides, and hydroxyl radicals, in the epidermis [15]. ROS disturb cellular anti-oxidative defense systems and induce abnormal DNA changes, thus contributing to a breakdown of skin homeostasis [16]. In this study, we showed that tectorigenin inhibits UV-B-induced cell death and intracellular ROS generation in keratinocytes.

Catalase and GSH are essential enzymes that protect the skin against UV-B-induced damage. Catalase breaks down hydrogen peroxide, rendering it nontoxic [17]. The overexpression of catalase was shown to protect keratinocytes by decreasing intracellular ROS, inhibiting apoptosis in keratinocytes [18], and reducing MMP-1 levels in dermal fibroblasts [19]. GSH reduces oxidative molecules, including hydrogen peroxides and lipid radicals, by acting as an electron donor, which results in its conversion to GSSG. GSH counteracts the UV-B-stimulated overexpression of MMP-1 in human dermal fibroblasts and ameliorates UV-B-induced wrinkle formation in human skin [20,21]. Our results showed that the treatment with tectorigenin triggered the production of catalase and GSH enzymes, which were otherwise depleted by UV-B irradiation.

ROS induced by UV-B irradiation cause skin cell death, in part by hindering the functions of keratinocytes, such as moisture retention and the formation of a barrier against the external environment. By demonstrating that tectorigenin regulates the UV-B-induced expression of apoptosis-related proteins, specifically Bcl-2, Bax and caspase-3, our results suggest that tectorigenin intervenes in the UV-B-induced apoptotic cascade in keratinocytes.

On the other hand, there are some arguments that the apoptosis induced by UV-B is a process to eliminate cells whose DNA is damaged [22]. UV-B-stimulated DNA damage in keratinocytes appears as the formation of pyrimidine dimers, such as cyclobutane pyrimidine dimers and 6-4 photoproducts [23], which are the primary cause of carcinogenesis in the skin [24]. Activated p53, a tumor suppressor gene, induces apoptosis of cells with damaged DNA in order to inhibit the proliferation of abnormal cells [25]. In this study, we established the inhibitory effects of tectorigenin on UV-B-induced apoptosis, but the possibility that tectorigenin induces carcinogenesis still remains. Moreover, some reports have revealed that HaCaT cells are less sensitive than primary keratinocytes in displaying a full apoptotic response because the p53 gene in the cell line is mutated [26]. Thus, to investigate that possibility, a further study should be conducted to measure the effects of tectorigenin on the formation of pyrimidine dimers and p53 expression in UV-B-irradiated primary keratinocytes.

UV-B-induced ROS also stimulate activator protein-1 and, thus, increase the levels of MMP-1, which mediate the breakdown of collagen types 1, 2, and 3. The resulting disruption of the extracellular matrix by MMP-1 contributes to skin wrinkling. Accordingly, MMP-1 may be an effective target for preventing photoaging [27]. Our results showed that tectorigenin protects keratinocytes, in part by inhibiting the elevated MMP-1 levels induced by UV-B.

In flavonoid compounds, the presence of hydroxyl groups and the low methylation ratio of the B-ring contribute to the anti-oxidative effects of these compounds [28,29]. Tectorigenin has three OH groups, at R5, R7, and R4’, and a methoxy group at R6. These structural features suggested its anti-oxidative effects and its ability to inhibit UV-B-induced skin photoaging, as reported for the soy isoflavones daidzein, genistein, and glycitein. The latter were previously shown to attenuate UV-B-induced cell death and pathological changes in human keratinocytes and mouse skin [30]. These features may have contributed to the anti-oxidative effects of tectorigenin.

## 5. Conclusions

The results of this study suggest the utility of tectorigenin as a potent antioxidant and, thus, as an inhibitor of skin photoaging, by attenuating UV-B-induced apoptosis and collagen degradation in human keratinocytes.
